# Critical Period of Memory Enhancement during Taste Avoidance Conditioning in *Lymnaea stagnalis*

**DOI:** 10.1371/journal.pone.0075276

**Published:** 2013-10-03

**Authors:** Tomoyo Takahashi, Satoshi Takigami, Hiroshi Sunada, Ken Lukowiak, Manabu Sakakibara

**Affiliations:** 1 School of High-Technology for Human Welfare, Tokai University, Numazu, Shizuoka, Japan; 2 Graduate School of High-Technology for Human Welfare, Tokai University, Numazu, Shizuoka, Japan; 3 Graduate School of Bioscience, Tokai University, Numazu, Shizuoka, Japan; 4 Hotchkiss Brain Institute, Faculty of Medicine, University of Calgary, Calgary, Canada; Alexander Fleming Biomedical Sciences Research Center, Greece

## Abstract

The present study investigated the optimal training procedure leading to long-lasting taste avoidance behavior in *Lymnaea*. A training procedure comprising 5 repeated pairings of a conditional stimulus (CS, sucrose), with an unconditional stimulus (US, a tactile stimulation to the animal’s head), over a 4-day period resulted in an enhanced memory formation than 10 CS-US repeated pairings over a 2-day period or 20 CS-US repeated pairings on a single day. Backward conditioning (US-CS) pairings did not result in conditioning. Thus, this taste avoidance conditioning was CS-US pairing specific. Food avoidance behavior was not observed following training, however, if snails were immediately subjected to a cold-block (4°C for 10 min). It was critical that the cold-block be applied within 10 min to block long-term memory (LTM) formation. Further, exposure to the cold-block 180 min after training also blocked both STM and LTM formation. The effects of the cold-block on subsequent learning and memory formation were also examined. We found no long lasting effects of the cold-block on subsequent memory formation. If protein kinase C was activated before the conditioning paradigm, snails could still acquire STM despite exposure to the cold-block.

## Introduction

Our understanding of the causal neuronal mechanisms that underlie learning and the subsequent formation of long-lasting memory has been greatly advanced by studies of simpler invertebrate model systems. Here we investigated the critical time periods following classical conditioning training during which memory formation can be interrupted using a reversible cold-block technique (cooling-induced amnesia). Knowledge of the critical time periods for consolidation is crucial to elucidate the molecular events that underlie memory.

To establish taste avoidance conditioning (TAC), we paired sucrose application (conditional stimulus, CS), which elicits a feeding response, with a tactile stimulus to the head (unconditional stimulus, US), which evokes a withdrawal response. The withdrawal response is incompatible with feeding, and thus the feeding response elicited by the CS is immediately terminated. After a number of CS-US pairings, the CS no longer elicits feeding. If the order of the pairing is reversed (i.e., US-CS; backward conditioning), the CS continues to elicit feeding behavior [1].

Cooling to less than 5 °C for a short period of time (tens of minutes) reversibly blocks the formation of long-term memory (LTM) following conditioning if performed within a short period of time following training [2–7]. This cooling procedure is hypothesized to block the consolidation process due to its interference with the metabolic processes necessary for LTM formation (i.e., new protein synthesis and altered gene activity).

The canonical view of memory formation is that learning is a serial process beginning with short-term memory (STM), which persists for minutes, followed by intermediate-term memory (ITM), which persists for a few hours, and then LTM, which persists for days to week to years [8]. STM does not require protein synthesis, whereas both ITM and LTM require protein synthesis, and LTM additionally requires altered gene activity [9–16]. The time at which ITM and LTM respectively are thought to form was used to guide us to apply the cold-block (see Methods) to determine if we could block memory formation.

Studies performed in both mammalian (human and rodent) and invertebrate (e.g. *Drosophila, Aplysia*, *Lymnaea*, *Apis*) preparations have demonstrated that ‘spaced’ training results in a longer lasting LTM than does ‘massed’ learning [17–23]. We have previously shown in *Lymnaea* that spaced or distributed training was more effective than massed training in both classical conditioning [24,25] and operant conditioning [21]. Here we determined whether spaced training was also more effective than massed training to result in enhanced memory in TAC.

In *Aplysia*, ITM requires both new protein synthesis and the persistent activation of protein kinase A (PKA). LTM requires both new protein and mRNA synthesis, which occurs following prolonged activation of PKA induced by the training procedure that produces LTM. The prolonged activation of PKA causes it to translocate from the cytoplasm of the neuron to the nucleus. In the nucleus, PKA phosphorylates cyclic AMP response element binding protein 1 (CREB-1). CREB-1 is a transcription factor, and its phosphorylation stimulates mRNA synthesis. Downstream from CREB-1 are the immediate response genes, including the CCAAT box enhancer-binding protein. Activation of the immediate response genes, in turn, stimulates the transcription of downstream genes that trigger long-term structural changes that are thought to be the morphologic correlates of LTM [26–31]. A similar dependence on CREB-1 for LTM formation has been demonstrated in *Lymnaea* [32].

A more recent study using a similar training procedure implicated the involvement of a molluscan insulin receptor in memory formation in *Lymnaea* TAC [33]. PKC may be better able to activate CREB leading to LTM formation than PKA because PKC potently activates CREB along with other pathways (including HUD or mRNA stabilizing pathway) regulating synaptogenesis [34,35]. For more than two decades, PKC activation has been implicated in associative memory formation in a variety of species, including the mollusk, *Hermissenda crassicornis*, rodents, and rabbits [36–39]. In *Hermissenda*, the PKC modulator, bryostatin (Bryo) enhanced STM by pathways involving PKC-initiated membrane protein phosphorylation [40,41]. In addition, protein synthesis specifically involving PKC activation by low-dose exposure to Bryo (<1 nM) also enhanced STM formation in visuo-vestibular conditioning in *Hermissenda* [40,42–44]. Here we examined whether the PKC activator Bryo enhanced *Lymnaea* TAC memory.

Here we first examined whether spaced training is more effective than massed training for TAC. Although we hypothesized that space training would be more effective, taste aversion may be differentially affected because in some taste aversion training procedures, the CS and US can be separated by hours and still be effective (i.e., the Garcia effect; Garcia 1974) [45]. Second, we investigated whether a cold-block applied immediately after conditioning disrupts TAC. Third, we examined whether there are other critical periods after conditioning during which a cold-block effectively disrupts memory formation. Fourth, we evaluated whether the effect of cooling is really reversible, i.e., does application of the cold-block prevent snails from learning and forming memory? Finally, we examined whether protein kinase C (PKC)-mediated phosphorylation is required to form STM.

## Material and Methods

### Animals

Laboratory-reared fresh water pond snails, *Lymnaea stagnalis*, with shell lengths of 20-25 mm, were maintained at 22°C in well-aerated fresh water on a 12-h light: 12-h dark cycle (on at 08:00). Snails larger than 20 mm in shell length are capable of classical conditioning [46]. Animals were maintained on a diet of cabbage and goldfish pellets until food deprived 24 h prior to experimentation. All the experiment was performed from November to February during which metabolic activity of snails was thought to be the least.

### Experimental apparatus for taste avoidance conditioning training

The Plexiglas experimental container (diameter: 60 mm and height: 20 mm) had a perfusion system with an inlet and outlet from which the solution inside the container could be entirely replaced within 30 s (rate: 250 ml/min). The container held 10 ml pond water in which snails were kept. The conditioning response, i.e., feeding response, was readily observed by a mirror placed under the container.

### Taste avoidance conditioning

The conditioning procedure used was identical to that reported by Kawai et al (2004). In brief, snails were allowed to acclimatize for 10 min at 22°C in well-aerated pond water in the training container. Following acclimatization, the feeding response (i.e., the number of mouth openings in bites/min) to the CS (sucrose) was recorded. The CS (1 ml of 100 mM sucrose) was applied directly to the lip of the animal with a 1-ml syringe. Immediately following the sucrose application, the number of bites per minute (‘feeding response’) was tabulated for 1 min. A tactile stimulus (US) was then applied to the animal’s head using a hand-held Plexiglas rod. The stimulation was strong enough to always evoke a whole-body withdrawal response, as well as termination of repetitive mouth opening and closing (feeding). This was the pre-conditioning test (pre-test). After 10 to 15 min, the snails received varying numbers of CS-US pairings separated by 5-s intervals applied using a 1-min intertrial interval. The snail typically required less than 1 min to recover from the US. An immediate post-conditioning test, was applied at least 10 min or later after conditioning, was performed following the paired CS-US presentations. In this test only the CS was delivered. The immediate post-conditioning test was used to determine whether STM had formed. We defined STM as being present if the feeding response after conditioning (i.e. the post-test) was significantly suppressed in comparison with pre-test response on that day. We also tested the response to the CS 24 h and 48 h after training. The 24-h and 48-h post-conditioning tests enabled us to determine whether LTM had formed. Our definition of LTM was that the feeding score in the 24 or 48 h post-test was significantly (*p*<0.05) lower than that of the initial pre-test, i.e. that of the first day pre-test. Backward conditioning with a tactile stimulus presented to the head (US) followed by sucrose application (CS) was performed to confirm whether TAC is CS-US temporal specific.

### Cold-block

A 500 ml beaker filled with well-aerated pond water was pre-chilled and maintained at 4 °C. This beaker served as the cooling apparatus. Snails were placed in the cooling apparatus for 10 min at various times after conditioning. In control experiments it was found that snails resumed eating behavior within 2 minutes of being placed into the test chamber maintained at room temperature (i.e. 22 °C). Thus, we always waited for about 10 min following their removal from the cooling apparatus before testing their response to the CS.

### Experiment 1-Spaced training vs. massed training and effectiveness of a cold-block

The first experiment was designed to determine whether 20 paired CS-US presentations given over 4 days resulted in better learning and memory formation than 20 paired CS-US presentations given over either 1 or 2 days.

We then examined whether the cold-block procedure interfered with memory formation. In the 4-day training procedure, snails were subjected to a cold-block immediately after the 5th CS-US pairing each day.

### Experiment 2-Memory consolidation process

Experiment 2 was designed to elucidate the critical time following training during which the cold-block prevents STM and LTM formation. We applied the cold-block at 4, 7, 10, 15, or 180 min after training. We then examined whether memory had formed by testing the response to the CS immediately after the cold-block and at 24 h and 48 h later. When the cold-block was applied with a 4-min delay, animals were placed into the chilled water for 10 min starting at 4 min post-conditioning. Following the cold-block snails were kept in the test chamber at room temperature (~22 °C) for at least 2 min before the “after-cooling immediate post-test” was performed.

**Figure 1:**
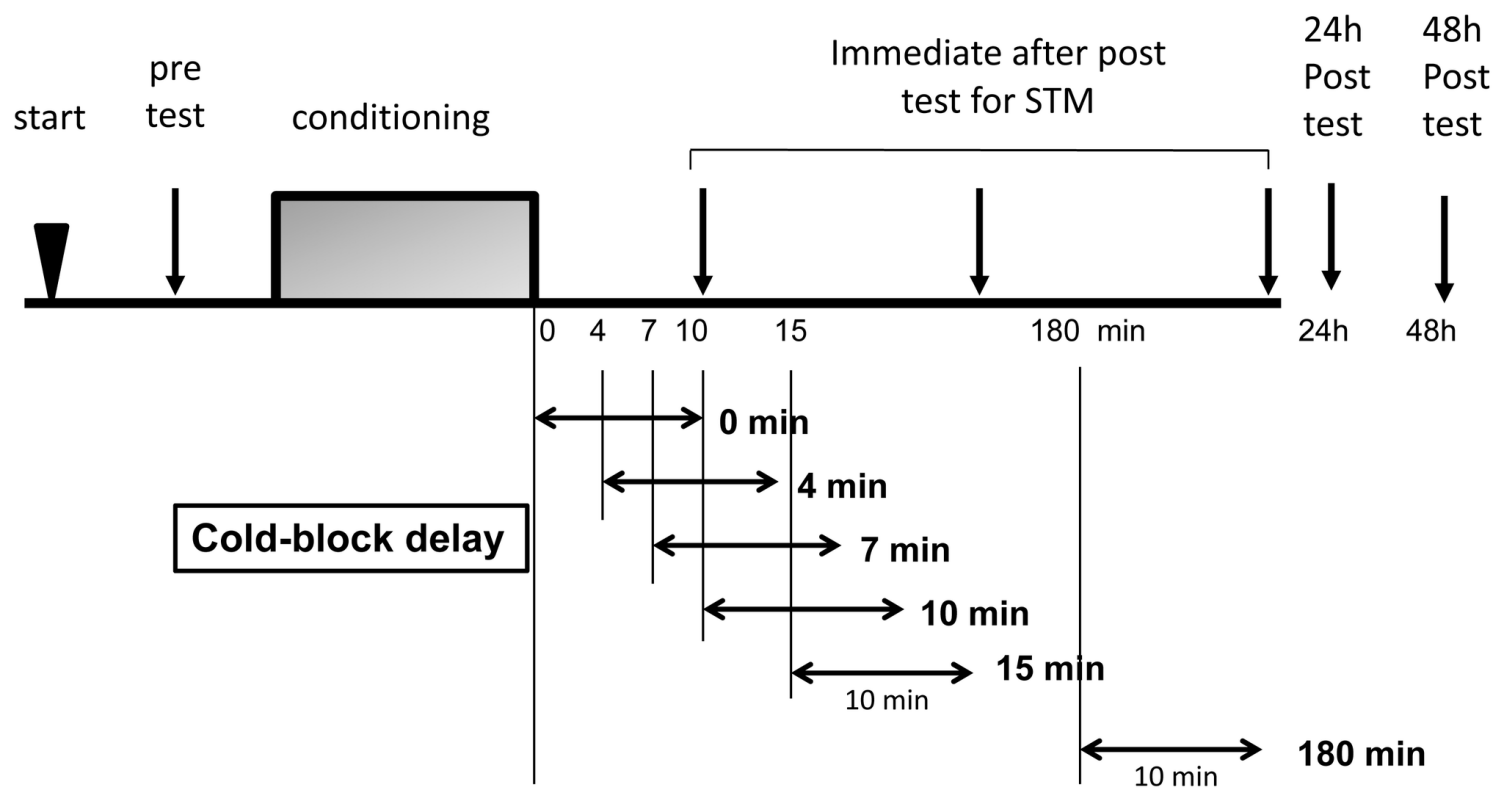
Experimental protocols for Taste Avoidance Conditioning (TAC) of *Lymnaea* *stagnalis* in a day. Snails were first adapted for 10 min prior to sucrose administration (CS) and pre-test feeding behavior was recorded (bites/min); then there was a 10-15 min recovery period followed by CS-sucrose/US-head touch paired associative conditioning trials (4, 10, or 20 pairings). Post-conditioning feeding responses were subsequently measured at ~10 min, after cooling, at 24 h, and at 48 h, and compared to the pre-test scores. Cold-block was applied immediately after conditioning (0-min delay), 4 min, 7 min, 10 min, 15 min, and 180 min, and the effect of the cold-block was evaluated by sucrose administration (CS). Short-term memory (STM) was evaluated by an immediate post-test. Long-term memory (LTM) was evaluated by a 24-h and 48-h post-tests.

Thus, each snail received the CS first as the pre-test, then the same number of CS-US pairings, the cold-block for 10 min at the various specified times following conditioning. In addition, they each were presented with the CS 3 times following conditioning at different times in order to determine if memory formed. The times were as follows: 1) immediately after the cold-block for STM; 2) 24 and; 3) 48 h after the conditioning for LTM. In the case of 15 min or 180 min experiments, we examined snails’ feeding response at 10 min after the final CS-US pairing as a post-test before subjecting the snails to the cold-block. To test the cold-block’s effect an immediate post-test was applied immediately after the cold-block. Thus, the post-test in 15 min and 180 min delay experiments was applied to snails twice (10 min and 27 min) after the final CS-US pairing. In experiment 2 we used 48 snails. The timing of each post-test session is shown in Figure 1.

### Experiment 3-Reversibility of cold-block

In this experiment, we examined whether cold-block-induced amnesia was reversible, in contrast to certain pharmacologic treatments. That is, we examined whether snails that were subjected to the cold-block procedure were still capable of forming LTM. For these experiments 7 snails were received 5 repeated pairings of the CS-US followed by an immediate cold-block, i.e., 0 min-delay, every day for 4 days. From the 5^th^ to the 8^th^ day these snails were again conditioned but without cold-block, i.e., they received 5 pairings of CS-US presentations for a further 4 days. They received a CS on the 5^th^ day (i.e. following the 4 days of pairing and cold block) prior to next 4 days of CS-US pairing without the cold block. They all received an immediate post-test on each of the next 4 days of pairing as well as a 24 h and 48 h post-test.

### Experiment 4-Involvement of PKC in STM formation

To test the hypothesis that STM formation is dependent on PKC activation, the PKC α and ε activator bryostatin was administered to the snails. Snails were placed in bryostatin-containing (0.5 ng/ml) pond water at 22°C for 45 min before the last day of training (5 CS-US pairings/day for 4 days). In these experiments, the cold-block was applied immediately after the final training session each day. STM and LTM were evaluated by an immediate post-test and a 24-h post-test, respectively.

### Bryostatin

The PKC activator bryostatin (LC Laboratories, Woburn MA) was initially dissolved in 100 µl ethanol and diluted in pond water to make a 1 µg/ml stock solution. This solution was further diluted in pond water to a final concentration of 0.5 ng/ml, with a final ethanol content of 0.005%. This was the maximum upregulating concentration of bryostatin found to be effective in the mollusk *Hermissenda* [40,44] and *Lymnaea* [47]. Animals in the experimental apparatus were immersed in a 10-ml water flow containing bryostatin (0.5 ng/ml) for 45 min. The bryostatin-containing water was then completely replaced (in 30 s) with fresh pond water.

### Statistics

Behavioral differences between pre- and post-conditioning (immediate, after-cooling post-test, 24 h, 48 h) were tested with repeated-measures analysis of variance (ANOVA). Scheffe’s *F* post hoc test was then used to further determine the statistical significance of the differences between groups by KaleidaGraph version 4.0 (HULINKS Inc., Tokyo, Japan). Both the behavioral measurements and the analyses were performed by researchers blind to the behavioral manipulation of the animals.

## Results

### Experiment 1-Spaced training vs. massed training and effectiveness of a cold-block

**Figure 2:**
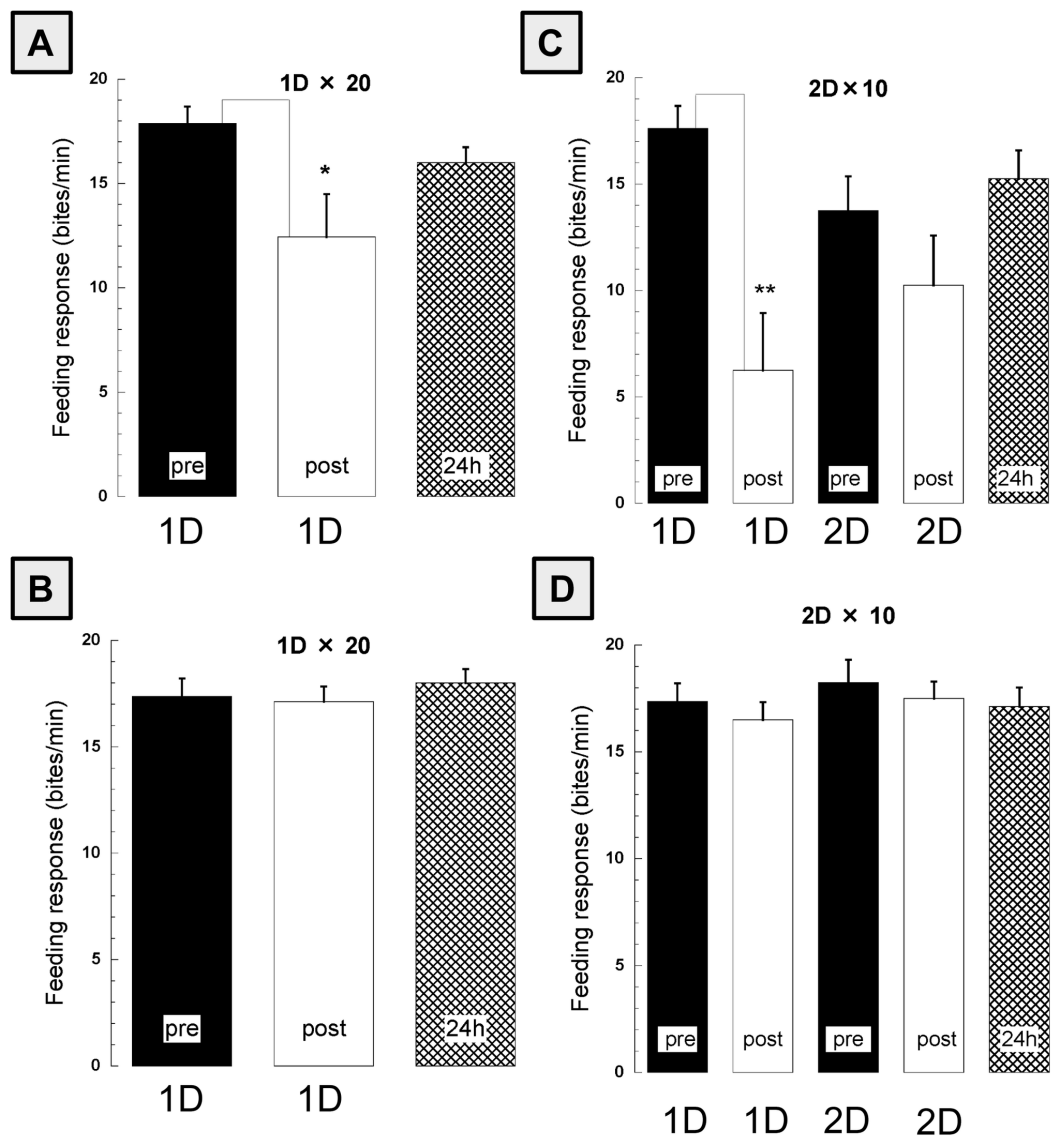
Cooling blocks memory formation. A) Snails (n=9) received 20 paired presentations of the CS-US on one day. These snails exhibited STM when tested 10 min after the last pairing (Post 1D, white bar), but did not exhibit LTM 24h later (checked bar). B) A second group of snails (n = 8) was trained as in A except the cold block was applied immediately after the last CS-US pairing. Both STM and LTM were blocked. C) A naive group (n = 8) received 20 CS-US pairings but the pairings were spread out over 2 days (i.e. 10/day). STM was observed but not LTM. D) As in C except that the cold block was immediately applied following the 10th CS-US pairing each day (n=8). Neither STM nor LTM formed. Each graph was composed of the scores of pre-test, 10 min post-test and 24-h post-test.

**Figure 3:**
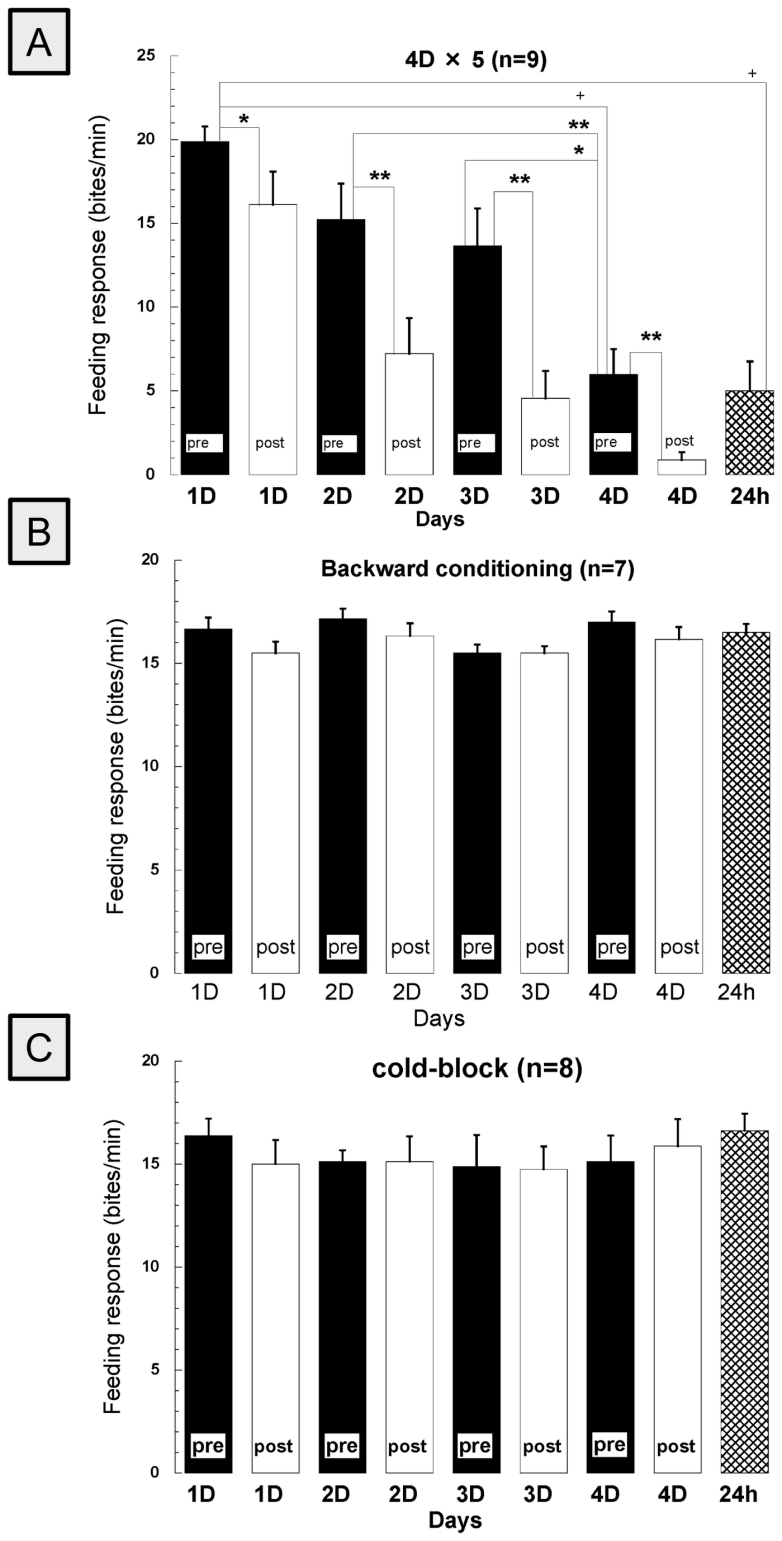
Dynamic changes in the feeding response of group 3 (4D×5 trials) each day (n=9) (A).No significant differences were detected after (B) backward conditioning, 5US+CS pairings for 4 days (n=7) or (C) after applying the cold-block immediately after conditioning each day (n=8). Values were compared to the pre-test value (1D pre) in Figure 3A if there was a significant main effect evaluated by repeated measures of ANOVA. Data are presented as mean ± standard error. *p <0.05, ** *p*<0.01, +*p*<0.0001.

We previously demonstrated that 20 paired CS-US presentations was the minimum number of pairings required for LTM formation [1]. Here we expanded on that report by investigating whether spaced vs. massed pairings of the CS-US (5/day for 4 days vs. 10/day for 2 days vs. 20 on a single day) resulted in stronger LTM. A total of 26 naive snails received 20 pairings of the CS-US. They were randomly divided into three separate groups: 1) 20 pairings in 1 day (n=9); 2) 10 pairings for each of 2 days (n=8); and 3) 5 pairings for each of 4 days (n=9). Following the 20 paired presentations in each of the groups, we evaluated both STM (later than 10 min after the last pairing) and LTM (24 h after the last pairing). For the evaluation, we compared the number of bites in the pre-test session in response to the CS with the number of bites elicited by the CS in the two memory test sessions. These data are presented in Figure 2A, 3A and Tables 1-3.

**Table 1:** Summary of the statistical significance of differences in feeding response among group 1(1D×20) in the pre-test, immediate post-test, 24-h post-test.

1D×20	pre	post	24h
pre		*	**NS**
post			**NS**

Data are test results for comparisons with the CS-US pairings of group 3(4D × 5), “pre” indicates the initial feeding response on day 1; “4D-post” indicates the response to the “immediate post-test” after the completion of 20 pairings; “24 h” indicates the response to the “24-h post-test” on the following day of conditioning.

NS: not significant; *p<0.05; **p<0.01; ***p<0.001; +*p*<0.0001

**Table 2:** Summary of the statistical significance of differences in feeding response among group 2(2D×10) in the pre-test, immediate post-test, 24-h post-test.

2D×10	1D pre	1D post	2D pre	2D post	24h
1D pre		**	**NS**	**―**	**NS**
2D pre				**NS**	**NS**

Data are test results for comparisons with the CS-US pairings of group 3(4D × 5), “pre” indicates the initial feeding response on day 1; “4D-post” indicates the response to the “immediate post-test” after the completion of 20 pairings; “24 h” indicates the response to the “24-h post-test” on the following day of conditioning.

NS: not significant; *p<0.05; **p<0.01; ***p<0.001; +*p*<0.0001

**Table 3:** Summary of the statistical significance of differences in feeding response among group 3(4D×5) in the pre-test, immediate post-test, 24-h post-test.

4D×5	1D pre	1D post	2D pre	2D post	3D pre	3D post	4D pre	4D post	24h
1D pre		*	**NS**	**―**	**NS**	**―**	**+**	**―**	**+**
2D pre				**	**NS**	**―**	**	**―**	***
3D pre						**	*	**―**	**
4D pre								**	**NS**

Data are test results for comparisons with the CS-US pairings of group 3(4D × 5), “pre” indicates the initial feeding response on day 1; “4D-post” indicates the response to the “immediate post-test” after the completion of 20 pairings; “24 h” indicates the response to the “24-h post-test” on the following day of conditioning.

NS: not significant; *p<0.05; **p<0.01; ***p<0.001; +*p*<0.0001

LTM were observed only in group 3, which received 5 pairings/day for 4 days (4D×5). That is, the number of bites in the pre-test session in response to the CS was significantly greater than the number of bites elicited by the CS in the 24-h post-test sessions (*p*<0.05). The 24-h post-test sessions in the other two groups were not significantly different, however STM was acquired in every groups (summary of the statistical significances are presented in Tables 1-3. Thus, STM was observed in every groups while LTM was only observed in one of the three groups, 4D×5.

We examined the effects of the cold-block procedure (Figure 2B, 2D and 3C) on the formation of STM and LTM in three groups (8 snails in each). Snails underwent the cold-block procedure after the last paired presentation of the CS-US each day. Thus, group 3 (4D×5), 8 snails underwent a total of four cold-block procedures, group 2 (2D×10) 8 snails underwent two cold-block procedures, and group 1 (1D×20) 8 snails underwent a single cold-block procedure. Neither STM nor LTM were observed in snails in any of the three groups as shown in Figure 2B, 2D and 3C.

Next, using the 5 pairings/day for 4 days training procedure, we examined the savings that occur on each subsequent day of training that ultimately leads to the establishment of LTM (Figure 3A). The number of bites during the immediate post-test each day was always less than the pre-test score, even if the decrease was not statistically significant. Further, the pre-test score on the following day (which is also a test for 24-h memory) was always, with the exception of day 2, higher than the 10-min post-test of the previous day. STM had appeared first on the first day and snails saved STM by the “every day conditioning paradigm” furthermore by day 4, LTM had formed. This saw-tooth decrease over the training days indicates that the snails had a day-to-day memory savings (i.e., the association between the CS-US was stronger each day) that eventually resulted in LTM formation by day 4. These data are tabulated in Table 3. The data shown in Figure 3B indicate that backward conditioning (i.e., US-CS pairing) does not prevent the CS from eliciting feeding. Finally, the cold-block procedure following the 5th presentation of the CS-US pairing prevented any savings from occurring over days and also prevented both STM and LTM formation (Figure 3C).

### Experiment 2-Memory consolidation process

**Figure 4:**
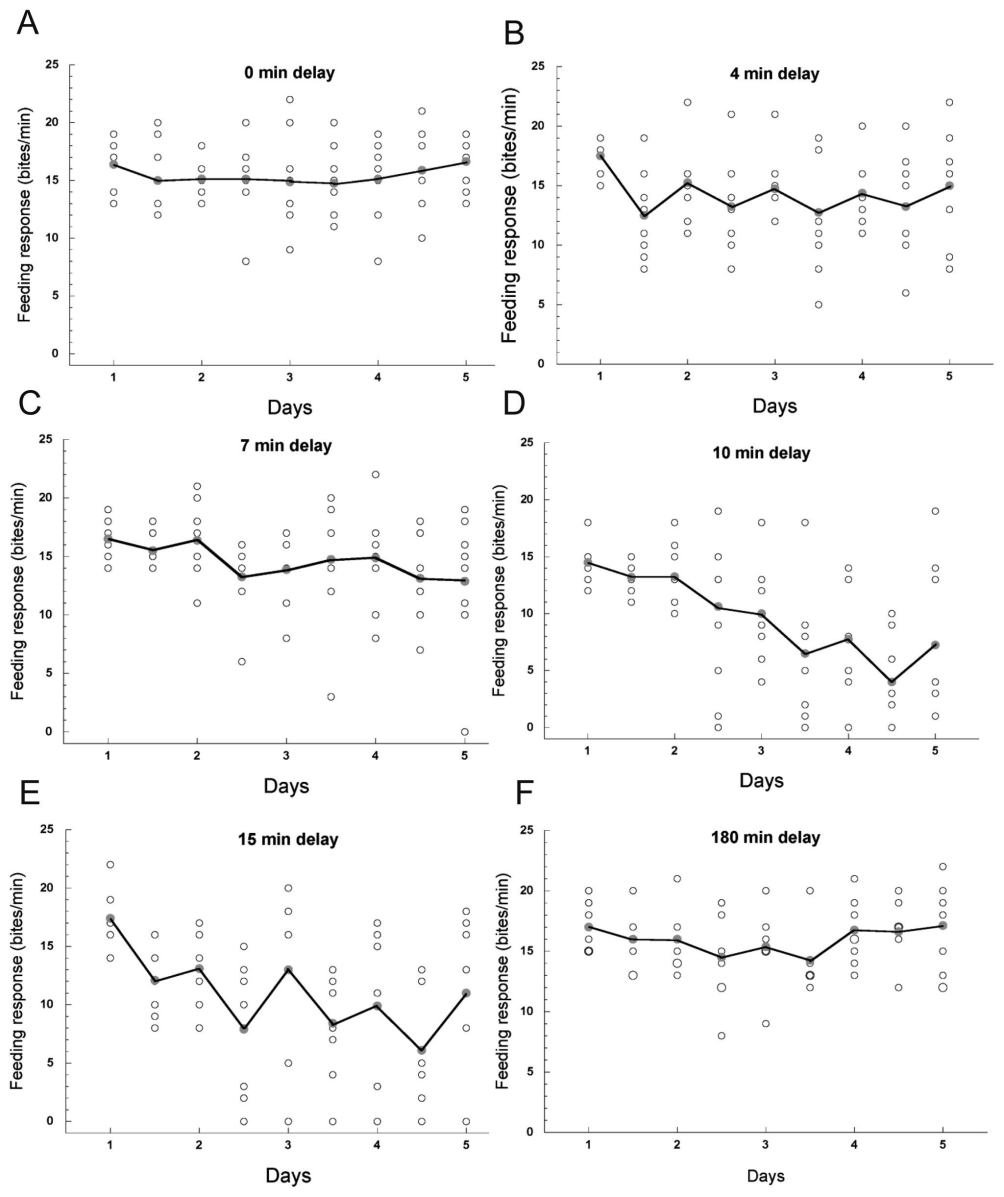
Effects of cold-block on the feeding response to the CS at various time points, (A) 0-min delay (n=8), (B) 4-min delay (n=8), (C) 7-min delay (n=8), (D) 10-min delay (n=8), (E) 15-min delay (n=8), and (F) 180-min delay (n=8). Conditioning was performed with 5 paired CS-US presentations for 4 days. Each scattered graphs were composed of 8 snails’ individual data, the mean of feeding scores was connected along with daily change. Within a day the scores of pre-test were shown first then immediate post-test scores were followed. The immediate post-test in 15 min and 180 min delay was applied to snails twice at 10 min and 27 min after the final CS-US pairing. In the cases snails received 15 min delay cold-block, they were examine to CS application twice before and after the cold-block, while 180 min delayed snails were tested before the cold-block twice. The scores of second immediate post-test in E and F were not included.

We first demonstrated that STM and LTM were blocked by subjecting trained snails to the cold-block procedure immediately after the last training session each day. Next, we set out to determine the critical time period in the memory consolidation process during which the cold-block procedure no longer disrupted memory formation. We did this by varying the time to apply the cold-block procedure after the last CS-US pairing each day (Figure 4). Each group of 8 snails experienced the same number of cold-block procedures, but the cold-block was applied immediately (0 min), 4, 7, 10, 15 or 180 min after the last pairing (Figure 1). The daily effects of cold-block on feeding behavior are shown in Figure 4. Each graph is made up of both the mean response and individual response of each snail. On each day the pre-test score is plotted first and then the immediate post-test scores follow. The scores of the second immediate post-test applied 27 min after the last CS-US pairing in Figures 4E and 4F are not included. The groups receiving the 4 and 7 min delayed cold block exhibited STM but not LTM (4min delay: 1D-pre vs. 1D-post *p*=0.0084, 2D-pre vs. 2D-post *p*=0.0035; 7 min delay: 2D-pre vs. 2D-post *p*=0.0063). The groups receiving the cold block 10 and 15 min after the final pairing showed both STM and LTM formation (Tables 4, 5). Also note that in Figure 4D and E, the feeding score tended to decrease day by day slowly compared to Figure 3A. Finally, LTM was not formed when the cold-block was given 180 min after the last pairing.

**Table 4:** Summary of the statistical significance of differences in feeding response among the cold-blocks of 10 min delay.

10 min delay	1D pre	1D post	2D pre	2D post	3D pre	3D post	4D pre	4D post	24h
1D pre		*	**NS**	**―**	*	**―**	*	**―**	*
2D pre				**NS**	*	**―**	**NS**	**―**	**NS**
3D pre						*	**NS**	**―**	**NS**
4D pre								**NS**	**NS**

NS: not significant; *p<0.05

**Table 5:** Summary of the statistical significance of differences in feeding response among the cold-blocks of 15 min delay.

15 min delay	1D pre	1D post	2D pre	2D post	3D pre	3D post	4D pre	4D post	24h
1D pre		*	*	**―**	**NS**	**―**	*	**―**	**NS**
2D pre				**NS**	**NS**	**―**	**NS**	**―**	**NS**
3D pre						**NS**	**NS**	**―**	**NS**
4D pre								**NS**	**NS**

NS: not significant; *p<0.05

So far, we demonstrated that both STM and LTM formation was prevented if the cold-block procedure was initiated without delay of the last CS-US pairing, i.e., 0 min delay. With 10-min delay STM was formed by day 1 and LTM by day 3. Following a 15-min delay after the last CS-US pairing, STM was observed on the first day. LTM, however, did form by day 2 as shown in Tables 4 and 5. We concluded, therefore, that delayed application of the cold-block procedure for 10 min or 15 min following each day’s CS-US pairing allowed for the formation of both STM and LTM. The results among 6 different delay on STM and LTM were summarized in Table 6. The decreasing tendency in feeding response to CS was obvious in Figure 4D and 4E; STM formation was characterized in saw-tooth decrease over the training days; LTM formation was characterized in approaching the mean value to zero but took time in comparison with that without cold-block.

**Table 6:** Summary of the cold-block delay on the formation of STM and LTM.

Delay (min)	STM	LTM
0	**×**	**×**
4	**1D, 2D**	**×**
7	**2D**	**×**
10	**1D**	**3D, 4D, 5D**
15	**1D**	**2D, 4D**
180	**×**	**×**

×: no memory formation; 1D: appearance at the first day; 2D: appearance at the second day; 3D: appearance at the 3rd day; 4D: appearance at the 4^th^ day; 5D: appearance at the 5^th^ day

The results of the presentation of the cold-block procedure to the snails at 180 min following the last pairing each day are shown in Figure 4F. To our surprise, neither STM nor LTM was formed on any day, and the variation of data was smaller than the other conditions in Figure 4. Thus, we conclude that delaying the cold-block procedure for 180 min following the last pairing of the CS-US each day was sufficient to block both STM and LTM formation.

### Experiment 3-Reversibility of the cold-block

**Figure 5:**
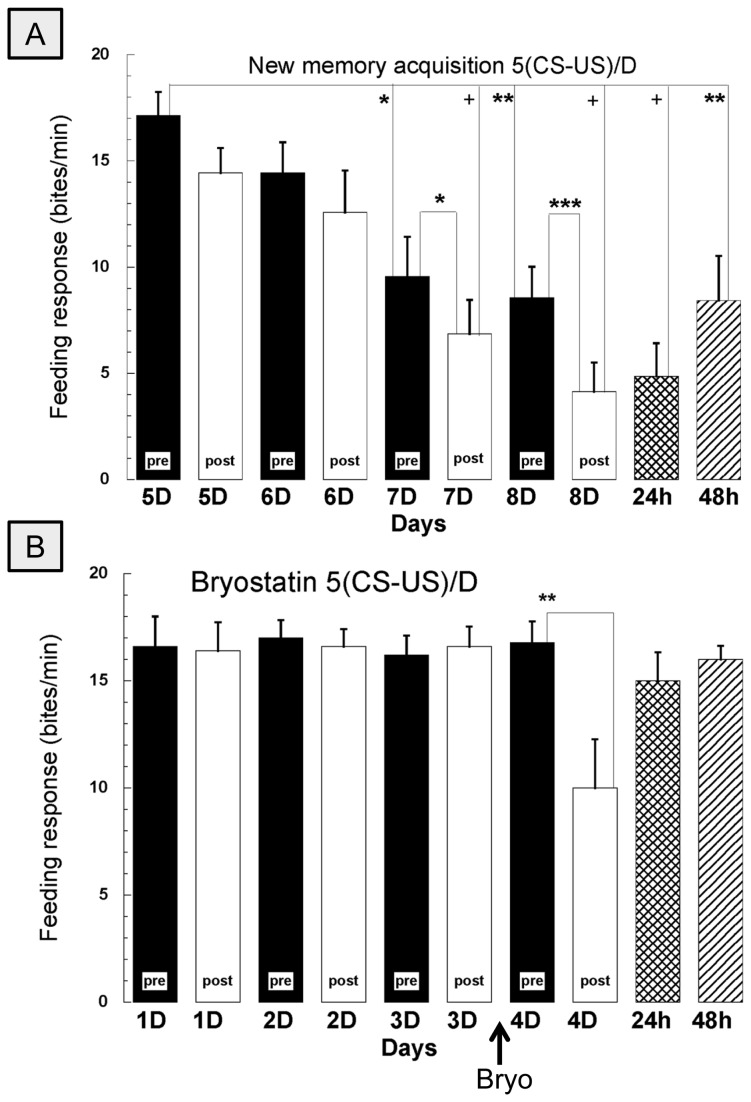
Taste avoidance conditioning was reversible (A) after immediate cold-block, and (B) during the amnesic state when PKC activation resulted in STM on the final day of conditioning. **A**) Snails experienced 0-min delay cold-block after conditioning for 4 days, and were then conditioned without cold-block for the next 4 days (n=7). Note that the learning kinetics to obtain the new memory formation were similar to those shown in Figure 3A. B) Snails did not exhibit memory when the cold block was applied immediately following the CS-US pairing (i.e. days 1-3). However, on day 4 before the training procedure snails were exposed to the PKC activator bryostatin for 45 min, and then received the 5 CS-US pairings in ‘normal’ water. This resulted in STM but not LTM formation (n=5). Note that 5 paired CS-US presentation was insufficient to cause STM in Figure 3A. Bryo represents the time to soak animals in bryostatin containing water on day 4 before the conditioning paradigm in the horizontal axis. * *p*<0.05, **p<0.01, ***p<0.001, +*p*<0.0001.

In this series of experiments, we investigated whether snails that underwent the cold-block procedure to induce amnesia could still learn and form memory. In these experiments (Figure 5A), 7 snails that had received 5 CS-US pairings for 4 days and that had received the cold-block immediately following the last pairing on each day were again trained for another 4 days, but without the cold-block. These snails exhibited STM and LTM on the expected days. Animals that had been unable to form STM because of the cold-block were now able to form both STM and LTM. Thus, the cold-block, while able to block both STM and LTM when applied at specific times, did not cause an irreversible blockage of learning and memory formation. Also, the rate of learning and memory in these snails appeared to be no faster than that in naive snails (see Figure 3A) despite having received previous training, albeit followed by the cold-block each day.

### Experiment 4-Involvement of PKC in STM formation

To test whether the conditioned memory was enhanced following PKC activation, we used bryostatin, a PKC activator, following training and cold-block application. Thus, a group of 6 snails received 3 days of training (5 pairings of the CS-US followed immediately by the cold-block each day). Neither STM nor LTM was observed. Before beginning the day 4 training, we placed the snails in bryostatin-containing pond water at 22 °C for 45 min and then proceeded to train the snails (5 CS-US pairings). This training procedure should not have resulted in STM, however STM (significant feeding suppression after the 5 pairings within a day) was observed even though the cold-block was applied immediately after the 5^th^ CS-US pairing on day 4 of training as shown in Figure 5B.

## Discussion

In this study we examined 110 *Lymnaea*; 57 snails for Experiment 1; 40 snails for Experiment 2; 7 snails for Experiment 3; 6 snails for Experiment 4. The findings of the present study confirmed that: 1) Spaced learning for TAC is more effective for inducing the formation of LTM; 2) A cold-block procedure prevents the formation of both STM and LTM if applied without delay of the last CS-US pairing on each day’s training; 3) A cold-block procedure prevents the formation of LTM if applied within 10 min of the last CS-US pairing on each day’s training; 4) A time delay of 180 min following the last CS-US pairing each day leads to no memory saving ; 5) The cold-block procedure, while sufficient to block both STM and LTM formation, did not permanently prevent snails from learning and forming both STM and LTM; and 6) PKC activity appeared to enhance memory formation.

### Mechanisms of LTM formation

Memory can be parsed into three categories based on its duration and molecular underpinnings. STM persists for minutes, ITM persists for a few hours, and LTM persists for days to months to years. STM does not require new protein synthesis whereas both ITM and LTM require new protein synthesis. In addition, LTM requires altered gene activity [9,48–54]. Altering the molecular processes following learning during the consolidation period blocks the formation of memory. A critical step in the consolidation process leading to long-lasting forms of synaptic plasticity and LTM is the activation of immediate early genes such as activity-related cytoskeleton association protein, early growth response gene 1, CCAAT/enhancer binding protein, *c-fos*, *c-jun*, and the recently identified target gene insulin-like growth factor 2 [30,55–60].

One of the known immediate early genes cloned and sequenced in TAC in *Lymnaea* is CCAAT/enhancer binding protein [32] and recent findings by Murakami et al. (2013) revealed that molluscan insulin-related peptide is involved in consolidation of TAC in *Lymnaea* [33].

Protein synthesis is essential for LTM formation. Two periods of sensitivity are assumed; one occurring immediately after training [15] and the other occurring several hours later [42]. In *Lymnaea*, Fulton et al (2005) reported that one-trial appetitive conditioning data suggest that there is a single period of sensitivity for the protein synthesis necessary for LTM that lasts from between 10 min to 1 h after training, based on studies using pharmacologic agents (the translation inhibitor anisomycin and the transcription blocker actinomycin D) [61]. Fulton et al (2008) showed the quite contrast results with the same appetitive conditioning that a cold-block procedure disrupted LTM only when applied immediately after conditioning, whereas delaying the treatment by 10 min left the 24-h memory trace intact [2]. Pharmacologic treatments, such as those used in the Fulton studies of 2005, have long-lasting irreversible effects on *Lymnaea* [3]. As demonstrated here, the cold-block induced amnesia was totally reversible.

Cooling snails immediately (i.e., within 30 s) after operant conditioning training, reactivation of memory, or extinction training blocks the consolidation or reconsolidation process and does not adversely affect the health of the snails [3,62,63]. In the present study, to block LTM formation, the cold-block had to be applied immediately within 10 min following the last CS-US presentation. Cooling snails immediately after the first bout of ITM training also prevents the establishment of the residual molecular memory trace [64]. A cold-block procedure similar to the one used here also prevented LTM formation following a one-trial training procedure that focused on aerial respiratory behavior [65]. In addition, administration of ketamine immediately after aerial respiratory one-trial training also blocks LTM formation [66]. Further, ketamine application as late as 120 min but not as late as 180 min blocks LTM formation following a one-trial training procedure. Thus, consistent with our findings, there are different periods where memory formation is susceptible to blocking. It was hypothesized in the Browning study that ketamine acts by blocking the altered gene activity necessary for LTM formation [66].

Thus, our findings using the cold-block procedure are consistent with the previous findings [2,3]. Here, we also determined the effect of the cold-block on STM, which was not evaluated in the previous studies utilizing a cold-block procedure. We demonstrated that the formation of both STM and LTM was blocked by cooling immediately after conditioning. The group overall at certain time points did statistically exhibit STM (4, 7, 10, and 15 min delay), but not meet LTM formation in 4 and 7 min delay. We therefore believe that the process of consolidation may proceed at earlier period by 10 min. We thought that the effect of the cold-block was due to cold-block induced halting or slowing down of the metabolic processes and/or macromolecular synthesis necessary for LTM. Actually this cooling induced slow down macromolecular process was apparent comparing with Figure 4F and Figure 3A suggesting the protein synthesis for LTM formation may be interfered by a long delayed cold-block.

To further complicate matters, we found that applying the cold-block 180 min after the last CS-US pairing each day blocked memory formation entirely, which was an unexpected finding. It could be argued that STM was not observed because once formed it only persisted a short period of time and had dissipated prior to testing 180 min after the last CS-US pairing and after the cold-block procedure. Why LTM was not apparent on the last day following this procedure remains an important question because it appears that the consolidation period should have been completed within 10 min after each day’s training. A somewhat similar phenomenon was recently been reported in *Lymnaea*, and in *Hermissenda* and *Aplysia*. In the *Lymnaea* study, which examined appetitive one-trial food conditioning, memory could be demonstrated at 10 min, 1 h, and 3 h, but not at 30 min or 2 h after a single training episode [67]. Moreover, the authors demonstrated that memory could be perturbed by a tactile stimulus, which initiated the whole snail withdrawal response, when applied 30 min and 2 h after the training episode. These memory lapses and susceptibility corresponded to transitions between different phases of memory with different molecular requirements. In the *Hermissenda* study by Crow and Xue-Bian, changes in the protein levels at two different times following one-trial *in vitro* conditioning were examined using two-dimensional difference gel electrophoresis. Significant protein regulation was detected at 30 min and 3 h post-conditioning. These proteins were involved in diverse cellular functions, such as translational regulation, cell signaling, cytoskeletal regulation, metabolic activity, and protein degradation [68]. In the *Aplysia* studies, memory at the behavioral level and its synaptic correlates were not observable at various times after training [69,70]. Interestingly, the Botzer et al. study also examined feeding aversion [69]. In their study, LTM only appeared 12 to 24 h after training. Memory was not apparent between 4 and 12 h after training. These authors also used a cold-block procedure that differed from ours in that it involved placing animals in a freezer, but the behavioral data do not radically differ from what we observed here. Cooling in the aforementioned *Aplysia* study, however, did not block STM as it did here. Finally, following a one-trial training procedure for operant conditioning of aerial respiration, ketamine blocked LTM formation if applied either immediately or 2 h after training [66]. The authors hypothesized that ketamine had this effect due to its ability to alter gene activity.

Finally there is another possibility. We cannot rule out the possibility that the experimental procedure used in the “180 min delay cold-block” induced extinction of the learned response. With the “180 min delay cold-block” the CS alone was applied twice (10 min and 27 min) after the last CS-US in order to test for STM before the cold-block. The extra CS application may initiate an extinction process and this process might be enhanced during a cold-block period, thus no LTM was observed.

### Mechanisms of STM formation

At the cellular level, memory is evidenced by a change in synaptic activity at the pre-and/or post-synaptic neurons [27,71,72]. Changes in intrinsic membrane properties, however, also contribute to memory formation [73,74].

In *Hermissenda*, a mollusk related to *Lymnaea*, classical conditioning (a photic stimulus paired with a rotational stimulus) results in a change in the excitability of type B photoreceptors due to long-lasting K^+^ channel inactivation [75,76]. In *Hermissenda*, bryostatin enhances memory formation via a PKC pathway [77]. Here we also demonstrated that bryostatin enhances memory formation in *Lymnaea* as shown in Experiment 4. These data are consistent with data from operant conditioning studies in *Lymnaea* showing that bryostatin enhances long-lasting memory formation [47]. Future studies in *Lymnaea* should focus on examining changes in the excitability of neurons such as RPeD11 [78–80] to determine whether the changes in excitability induced by bryostatin are required for LTM formation.

The data presented here are also consistent with the finding reported by Epstein et al. in 2003 that the protein synthesis required for ITM and LTM occurs within 15 min following conditioning and again after 60 min, suggesting that a second round of protein synthesis is required for memory following classical conditioning in *Hermissenda* [42]. Here we showed that memory formation could be disrupted by applying a cold-block 180 min following completion of the conditioning. Thus, it appears that different periods of protein synthesis underlie the different forms of memory (e.g., ITM and LTM) following training. These findings are in contrast with the previous simpler suggestion that a single period of protein synthesis occurring immediately following conditioning underlies memory formation.

## Conclusion

*Lymnaea* were successfully conditioned with 20 paired presentations of a sucrose stimulus as the CS and a tactile stimulus to the head as the US. The most effective training procedure leading to LTM formation was a ‘spaced’ procedure, comprising 5 paired CS-US presentations each day for 4 days. This memory persisted for more than 1 week. Application of a cold-block immediately was sufficient to disrupt both STM and LTM. Delaying the cold-block procedure by 10 to 15 min allowed for the formation of STM and LTM. Delaying the cold-block by 180 min following the completion of training also blocked both STM and LTM formation, suggesting that there are two critical periods for LTM formation. The initial period may correspond to macromolecular protein synthesis while the 180-min period may correspond to mRNA transcription or a second wave of protein synthesis.
